# A Circular-Linear Probabilistic Model Based on Nonparametric Copula with Applications to Directional Wind Energy Assessment

**DOI:** 10.3390/e26060487

**Published:** 2024-05-31

**Authors:** Jie Liu, Zaizai Yan

**Affiliations:** College of Science, Inner Mongolia University of Technology, Hohhot 010051, China; liujiecando@163.com

**Keywords:** wind speed, wind direction, nonparametric kernel estimation, copula models, directional wind energy assessment

## Abstract

The joint probability density function of wind speed and wind direction serves as the mathematical basis for directional wind energy assessment. In this study, a nonparametric joint probability estimation system for wind velocity and direction based on copulas is proposed and empirically investigated in Inner Mongolia, China. Optimal bandwidth algorithms and transformation techniques are used to determine the nonparametric copula method. Various parameter copula models and models without considering dependency relationships are introduced and compared with this approach. The results indicate a significant advantage of employing the nonparametric copula model for fitting joint probability distributions of both wind speed and wind direction, as well as conducting correlation analyses. By utilizing the proposed KDE-COP-CV model, it becomes possible to accurately and reliably analyze how wind power density fluctuates in relation to wind direction. This study reveals the researched region possesses abundant wind resources, with the highest wind power density being highly dependent on wind direction at maximum speeds. Wind resources in selected regions of Inner Mongolia are predominantly concentrated in the northwest and west directions. These findings can contribute to improving the accuracy of micro-siting for wind farms, as well as optimizing the design and capacity of wind turbine generators.

## 1. Introduction

Wind power has gained significant traction in the global energy mix due to its clean and renewable nature. In order to promote the energy revolution and reduce carbon emissions, there is a strong focus on wind power engineering, which offers pollution-free characteristics and wide distribution advantages. International research and development efforts prioritize this field. During the “14th Five-Year Plan” period, China plans to add 310 million kilowatts of installed wind power capacity. According to statistics from China’s National Energy Administration (NEA), approximately 37.6 GW of newly installed wind power capacity was expected in 2022; Inner Mongolia leads in terms of installed capacity. It is imperative to accurately and reliably assess wind resources in order to combat climate change and ensure energy security [1]. Two important characteristics of wind are its speed and direction, with variations in wind energy density observed across different directions. Therefore, studying the joint probability density function (JPDF) of wind speed and direction allows us to quantify their correlation and assess the potential for wind energy associated with a specific wind direction [2,3,4]. Wind resource assessment in conjunction with wind direction is important for improving the accuracy of micro-siting for wind farms, reducing the operating costs, and improving the efficiency of wind turbines and power generation availability [5].

In recent years, various statistical models have been utilized to fit wind speed random variables. These include the Rayleigh and Weibull distributions [6], lognormal distribution [7], and generalized extreme value distributions [8]. D’Amico et al. [9] put forward an approach of modeling wind speed data through using a semi-Markov chain. Compared with a general Markov chain, the synthetic time series generated by this model can more accurately reflect the statistical characteristics of wind speed data, among which the second-order semi-Markov process of state and duration fits best. Aljeddani and Mohammed [10] used the probability density function (PDF) of the inverse Weibull distribution to model the wind speed characteristics. They proposed a modified maximum likelihood function based on this specific distribution to enhance parameter estimation accuracy, resulting in a reliable framework for wind speed assessment. Researchers have also investigated the potential to improve the validity and robustness of marginal PDF for wind speed by employing mixture distribution models [11,12], extended distribution models [13,14], and nonparametric kernel density estimations [15,16,17]. Alharthi [18] introduced a new statistical model called the modified sine-Weibull distribution. This model was used to analyze wind data from Spain by incorporating the Weibull distribution into the modified sine-G family of distributions. This approach represents a new advancement in utilizing trigonometric functions for wind speed modeling. In the modeling and prediction of wind direction, Hirata et al. [19] proposed a nonlinear multi-observation wind direction prediction model, which led to the improvement of prediction performance and expected power generation. Despite the extensive research on wind speed modeling, there is a scarcity of studies focusing on continuous wind angular probability distributions. Currently, the most commonly used distributions to characterize changes in wind direction are harmonic functions [20] and finite mixtures of von Mises distributions [21].

Previous findings suggest the limitations of assuming complete independence between wind speed and wind direction, and that the interdependence of the two variables should be fully considered. Johnson and Wehrly [22] proposed an angle-linear (AL) approach which describes variable dependency by defining circular-related coefficients. Carta et al. [23] improved the AL model and applied it to the study of wind speed and wind direction in JPDF. The wind speed marginal distribution for this JPDF model was described by a mixed Normal-Weibull distribution, and the marginal distribution of wind direction was obtained by fitting a mixed von Mises distribution. Since then, the AL model has become a representative method for constructing JPDFs of wind vectors, as it has better matching performance than conventional models [24,25]. In fact, in complex geographical areas where air can be blocked or accelerated, it can lead to strong winds in the prevailing direction. At this point, the AL method is restricted by symmetry and does not always adequately represent dependence structures.

Recently, the copula function has been widely used to construct joint models for multivariate random variables [26,27,28]. This approach allows for independently determining the marginal distributions without interference, offering high flexibility to capture non-normal and asymmetric distributional features [29]. A number of researchers have extensively explored the use of copula functions in wind energy studies, showcasing their ability to accurately describe the correlation among wind characteristics. An analysis of directional wind power generation in the German region was conducted by Schindler and Jung [30] using the Gaussian copula model. Li et al. [31] demonstrated that the copula approach is superior in binary distributions adjustment of wind speed and direction, as well as in predicting extreme wind speeds, through a comparison of its performance with that of conventional methods. Huang et al. [32] evaluated the directional wind energy potential in Hong Kong based on various copula functions.

However, it is worth noting that the studies mentioned above utilized parametric copula models, which rely on a priori distributional assumptions and are limited in terms of the types of distributions they can handle. These assumptions and limitations can introduce errors when applied to real data. A non-parametric kernel density estimator is a fully data-driven model, in contrast to parametric models. Without assuming a specific functional form, the model is capable of managing intricate relationships among variables. This ability allows it to effectively capture the non-linear correlations between variables, which provides a unique advantage. Charpentier et al. [33,34] proposed a non-parametric copula model utilizing kernel functions. Among them, the kernel-based copula model on the basis of the transformation idea was used to analyze financial risk data; the beta boundary kernel is optimally sophisticated and robust in analyzing wind speed and direction data [35]. The empirical Bernstein Copula (EBC) proposed by Sancetta and Satchell shows great flexibility in correlation analyses of circular–circular variables or circular–linear variables. Carnicero et al. illustrated the Bernstein Copula-based circular–linear and circular–circular modeling approaches using two cases, one of the relationship between wind direction and precipitation, and the other between the wind directions of two adjacent buoys [36]. In a recent study [37], the nonparametric Bernstein copula was used to construct a JPDF of wind speed and direction, where the order of the model was deter-mined by a stepwise search strategy combined with the cube root of the sample size recommended by Sancetta and Satchell. The model accurately describes the prevailing wind direction in complex wind environments and, in addition, the EBC method provides desired JPDF accuracy when the marginal distributions are poorly represented. However, up to the present, there have been few research studies on the performance of nonparametric copula methods for the fitting of JPDFs of wind speed and direction. Previous literature [30,38,39] contributed to the fields of wind speed and wind energy; however, they did not consider nonparametric models.

Situated along the northern border of China, Inner Mongolia boasts abundant wind resources. In order to promote balanced development and respond to the call for sustainable development, Inner Mongolia has become a key region for wind energy development in China. While many studies have analyzed wind speed variations and characteristics of wind energy distribution, there is a lack of research applying nonparametric copula methods to construct JPDFs for wind speed and direction in Inner Mongolia. Additionally, no study has explored the potential of directional wind energy in Inner Mongolia or its impact on engineering structures using this method. To address this gap, this study introduces a non-parametric copula model that utilizes a probabilistic transformation and optimal bandwidth algorithm to establish correlations between wind speed and wind direction. Various parametric copula models and models that do not consider interdependence are also introduced for comparison purposes. Measured data from monitoring stations in four similar allied cities in Inner Mongolia are utilized to evaluate the fitting accuracy of the various models; meanwhile, marginal PDFs of wind vectors suitable for this study area are obtained. Then, JPDFs of wind speed and direction are established on the basis of the nonparametric copula model, and subsequently, direction-dependent wind energy assessment is carried out.

The rest of this paper is structured as follows. Section 2 introduces the nonparametric methodology for constructing the marginal PDFs and binary JPDFs, as well as the model evaluation metrics. Section 3 briefly describes the wind data used. Section 4 compares the fitting accuracies of the different models and determines the JPDFs for wind speed and direction, as well as obtaining the marginal PDFs. Section 5 calculates the directional wind energy for sites located in four unallied cities in Inner Mongolia employing the superior JPDF model. Section 6 summarizes the entire paper.

## 2. Nonparametric Probabilistic Model

This section provides a brief description of the characteristics of the wind vector components considered in this study. Nonparametric kernel density estimation (KDE) models are established separately for wind speed and wind direction, yielding marginal probability density distributions for both. In this paper, we conduct a correlation analysis between wind speed and wind direction, introducing a nonparametric kernel density estimation copula (KDE-COP) model as well as several classical copula models developed for the JPDF of the wind vector. Subsequently, various evaluation metrics are introduced to evaluate the fitting performance of the models. Among them, the KDE model and the KDE-COP model employ an optimal bandwidth algorithm to select the most suitable bandwidth.

### 2.1. Marginal Probability Density Function of Wind Speed

When employing a kernel density estimation model, the initial challenge is selecting the appropriate kernel function and bandwidth. Based on historical research experience, the optimality of different choices of kernel functions in kernel density estimation is nearly consistent. In practice, the selection of the smoothing parameter (bandwidth, denoted as *h*) is a crucial and complex issue that directly impacts the performance of the kernel estimation. If *h* is chosen too small, the resulting kernel estimation curve exhibits pronounced fluctuations, and it may not be sufficiently smooth, which leads to an increase in variance. Conversely, if *h* is chosen too large, it may overlook the multimodality of the kernel estimation, resulting in an overly smooth curve and causing significant estimation bias.

For a sample $x_{1}$, $x_{2}$, …, $x_{n}$ from an unknown density $f\left(x\right)$, the kernel estimator expression for the wind speed probability density function is as follows:

(1) $$\hat{f}(x;h)=\frac{1}{n}\sum_{i=1}^{n}K_{h}(x-x_{i})$$

where *n* is the sample size, *h* is the bandwidth, and $K_{h}\left(u\right)=\frac{K\left(u/h\right)}{h}$, $K\left(\cdot \right)$ represents the kernel function. In this paper, the Gaussian kernel is chosen as the kernel function for fitting wind speed data in the KDE model, $K\left(u\right)=\frac{1}{\sqrt{2\pi }}\exp\left(-\frac{u^{2}}{2}\right)$.

The choice of bandwidth is typically made to minimize the error function, such as the Mean Integrated Squared Error (MISE) or Asymptotic expression for the MISE (AMISE). For the density function $f\left(x\right)$ and its corresponding kernel estimator $\hat{f}(x)$, the MISE can be expressed as follows:

(2) $$MISE\left(h\right)=E\left({\int \left[\hat{f}\left(x\right)-f\left(x\right)\right]}^{2}dx\right)\approx \frac{c(K)}{nh}+\frac{h^{4}d^{2}(K)c\left(f^{\prime \prime }\right)}{4}$$

in which $c(g)=\int g^{2}(u)du$, *g* gives a square-integrable function and $d(K)=\int u^{2}K(u)du$. The second-order continuous derivative of the target density is denoted by $f^{\prime \prime }$, and $f^{\prime \prime }$ proves square-integrable. Asymptotically optimal bandwidth by minimizing the MISE (2) can be obtained by

(3) $$h_{\mathrm{AMISE}}={\left(\frac{c(K)}{d^{2}(K)c\left(f^{\prime \prime }\right)}\right)}^{1/5}n^{-1/5}$$

Currently, it is common that the bandwidth selection methods include rule-of-thumb, plug-in (PI), and data-driven cross-validation (CV) methods. Applying the idea of the normal reference distribution rule (nrd0) [40] and from Equation (3), the expression for the optimal bandwidth is obtained by

(4) $$h_{nrd0}=0.9\hat{\sigma }n^{-1/5}$$

In the above equation, $\hat{\sigma }$ is taken as $\min\left\{S,Q/1.34\right\}$, in which *S* is the standard deviation of the sample and *Q* is the difference between the 75% and 25% quantile of the sample. By another rule of thumb, nrd [41], the factor in Equation (4) is taken to be 1.06 in the paper. This bandwidth formula is the adjusted Equation (4), i.e.,

(5) $$h_{nrd}=1.06\hat{\sigma }n^{-1/5}$$

The Least Squares Cross-Validation (LSCV) method, which automatically generates the optimal bandwidth from the data, produces an unbiased estimate of the bandwidth and is a commonly used method in solving for the bandwidth. Expanding the first equation in expression (2),

(6) $$MISE\left(h\right)=E\left(\int {\hat{f}}^{2}(x)dx-2\int \hat{f}(x)f(x)dx+\int f^{2}(x)dx\right)$$

It is evident that the last term in the above expression does not depend on $\hat{f}(x)$, and consequently, nor is it dependent on h. Therefore, the minimization formula is equivalent to minimizing

(7) $$R(h)=\int {\hat{f}}^{2}(x)dx-2\int \hat{f}(x)f(x)dx$$

According to the principles of LSCV, the following LSCV estimate can be constructed:

(8) $$LSCV(h)=\int {\hat{f}}^{2}(x)dx-\frac{2}{n}\underset{i}{\sum }{\hat{f}}_{-i}\left(X_{i}\right)$$

in which ${\hat{f}}_{-i}\left(X_{i}\right)=\frac{1}{\left(n-1\right)h}\sum_{j\neq i}K\left(\frac{X_{i}-X_{j}}{h}\right)$. Hence, the bandwidth estimation on the basis of the LSCV method is given by

(9) $$h_{LSCV}=\arg\underset{h>0}{\min}LSCV\left(h\right)$$

In addition to the aforementioned linear bandwidth selection algorithms, two algorithms introduced by Sheather and Jones using the plug-in method are also widely used. These two algorithms are known as the direct plug-in rule (SJ-dpi) and the solve-the-equation rule (SJ-ste), which utilizes the prior estimation of the derivatives to select the bandwidths. In this paper, this method can also be used in the bandwidth estimation of wind speed models.

The above kernel density estimation models with different bandwidths are recorded as KDE-nrd0, KDE-nrd, KDE-lscv, KDE-dpi, and KDE-ste, respectively.

### 2.2. Marginal Probability Density Function of Wind Direction

For an angular sample $\theta_{1},\theta_{2},\ldots ,\theta_{n}$ ∈[0,2π) from an unknown density f(θ), the circular kernel density estimator of f(θ) is defined as follows:

(10) $$\hat{f}(\theta ;\nu )=\frac{1}{n}\overset{n}{\underset{i=1}{\sum }}K_{\nu }\left(\theta -\theta_{i}\right),\;0\leq \theta <2\pi$$

where the bandwidth parameter is denoted by ν,ν>0, and $K_{v}$ represents the circular kernel function.

Currently, the most widely used parametric model for circular data is the von Mises distribution, which has a PDF of

(11) $$f_{vMs}(\theta ;\mu ,\kappa )=\frac{1}{2\pi I_{0}\left(\kappa \right)}\exp\left\{\kappa \cos(\theta -\mu )\right\}$$

where *θ* represents the wind angle, the scale parameter κ≥0, and μ∈[0,2π) denotes the mean value of wind direction. $I_{r}(\kappa )$ denotes the r-order modified Bessel function of the first kind. Taking into consideration the flexibility of the von Mises distribution, this paper employs the density function of the von Mises distribution as the kernel function in wind direction kernel density estimation, yielding the density estimator in view of the von Mises kernel as follows:

(12) $$\hat{f}\left(\theta ;\nu \right)=\frac{1}{n\left(2\pi \right)I_{0}\left(\nu \right)}\sum_{i=1}^{n}\exp\left\{\nu \cos(\theta -\theta_{i})\right\}$$

Here, ν represents the smoothing parameter (bandwidth) of the kernel density.

Following the principle of cross-validation, the optimal bandwidth can be solved by searching for the maximum value for the likelihood cross-validation (LCV) function, expressed as follows:

(13) $$LCV(\nu )=\overset{n}{\underset{i=1}{\prod }}{\hat{f}}_{-i}\left(\theta_{i};\nu \right)$$

In this equation, ${\hat{f}}_{-i}\left(\theta_{i};\nu \right)$ denotes the circular kernel density estimate excluding the ith observed value. Consequently, the maximum likelihood bandwidth for the circular kernel density is given by the following

(14) $$\nu_{LCV}=\arg\underset{\nu >0}{\max}LCV\left(\nu \right)$$

The mean integrated squared error for circular kernel density is represented by $MISE(\nu )=Ε\int {\left[\hat{f}(\theta )-f(\theta )\right]}^{2}d\theta$. The MISE typically lacks a closed-form expression, and practitioners often resort to optimizing its asymptotic approximation [42], AMISE of MISE is derived as

(15) $$AMISE(\nu )=\left\{\frac{1}{16}{\left[1-\frac{I_{2}(\nu )}{I_{0}(\nu )}\right]}^{2}\int_{0}^{2\pi }{\left[f^{\prime \prime }(\theta )\right]}^{2}d\theta +\frac{I_{0}(2\nu )}{2n\pi {\left(I_{0}(\nu )\right)}^{2}}\right\}$$

where $f^{\prime \prime }$ represents the second derivative of the target density to be estimated.

According to the well-known rule of thumb [43], the samples are assumed to obey a von Mises distribution with a scale parameter κ, which is used as a reference density for the target circular density f, in that way

(16) $$AMISE(\nu )=\frac{3\kappa^{2}I_{2}(2\kappa )}{32\pi \nu^{2}I_{0}{\left(\kappa \right)}^{2}}+\frac{\nu^{1/2}}{2n\pi^{1/2}}$$

Thus, the optimal bandwidth by minimizing the above equation is gained as

(17) $$\nu_{RT}={\left[\frac{3n{\hat{\kappa }}^{2}I_{2}(2\hat{\kappa })}{4\pi^{1/2}I_{0}{\left(\hat{\kappa }\right)}^{2}}\right]}^{2/5}$$

in which $\hat{\kappa }$ denotes the maximum likelihood estimate of the scale parameter κ.

Another approach is the plug-in rule, which is adopted in this paper to insert the mixture von Mises distribution in Equation (15) as the reference density [44]. A finite mixture of M von Mises distributions, $vonM\left(\mu_{i},\kappa_{i}\right)$, i=1,…,M is defined as

(18) $$g(\theta )=\overset{M}{\underset{i=1}{\sum }}\alpha_{i}\frac{\exp\left\{\kappa_{i}\cos\left(\theta -\mu_{i}\right)\right\}}{2\pi I_{0}\left(\kappa_{i}\right)}$$

In the above equation, $\alpha_{i}$ represents the weight coefficient, with $\sum_{i=1}^{M}\alpha_{i}=1$. After obtaining the estimate of *AMISE*(ν), the estimation of bandwidth $\nu_{PI}$ is calculated using minimization of *AMISE*(ν).

Among the numerous bandwidths solving algorithms used regarding kernel density estimation on cyclic data, data variability may give rise to unsolved cases. Ameijeiras-Alonso [45] conducted a new study by proposing the direct plug-in rule (AA-dpi) and the solving the Equation (AA-ste) rule based on the plug-in idea, which are bandwidth methods for circular data that can be iterated until the derivative estimation of the target density is obtained. It serves as an extension of the bandwidth provided by Sheather and Jones. The bandwidth estimates obtained in this paper by applying these two rules are denoted as $\nu_{DPI}$ and $\nu_{STE}$, respectively, and their algorithms are implemented in the R package NPCirc.

The circular kernel density estimation models with different bandwidths are respectively recorded as KDE-_LCV_, KDE-_RT_, KDE-_PI_, KDE-_DPI_, KDE-_STE_.

### 2.3. Metrics for Model Evaluation

In this paper, three metrics are introduced to assess the goodness-of-fit of the marginal distribution: the coefficient of determination (*R*^2^), the root-mean-square error (*RMSE*), and the mean absolute error (*MAE*). The expressions of these metrics are shown in Table 1.

In Table 1, $F_{i}$ and ${\hat{F}}_{i}$ represent the actual and the estimated values of the distribution for the *i*th sample, respectively; $\bar{F}$ denotes the mean of the modified empirical cumulative distribution, and *n* is the sample size. In the above metrics, a higher *R*^2^ approaching 1, and smaller *RMSE* and *MAE* values, indicate a better accuracy in model fitting.

### 2.4. Joint Probability Density Function Estimation of Wind Speed and Direction

For wind speed and wind direction variables, conventional linear coefficients may not accurately reflect the correlation between them. Copula functions, as a type of linking function, are widely applied in correlation analysis. In this paper, it can be used to characterize the non-linear relationship between the wind speed and wind direction bivariate variables. $u=F_{X}\left(x\right)$ and $v=F_{\Theta }\left(\theta \right)$ represent the cumulative distribution functions (CDFs) of the two wind vector variables, respectively, and their joint cumulative distribution function (JCDF) is denoted as *F* (*x*, *θ*). In accordance with Sklar’s theorem [46], the relationship between wind speed and direction could be expressed with a correlation function

(19) $$F(x,\theta )=C\left(F_{X}(x),F_{\Theta }(\theta )\right)$$

since $F_{X}\left(x\right)$, $F_{\Theta }\left(\theta \right)$$\in \left[0,1\right]$, then $C\in {\left[0,1\right]}^{2}\to \left[0,1\right]$. The JPDF of wind speed and direction is expressed as the following equation:

(20) $$f(x,\theta )=c\left(u,v\right)f(x)f(\theta )$$

Parametric models are typically used to estimate the probability distribution of the copula functions, and the commonly used parametric copula models include the Gaussian copula, Student t-copula, Clayton copula, Frank copula, and Gumbel copula. The formulas of these models can be found in the literature [31]. However, parameter copula models also have limitations, such as exhibiting boundary biases and being more suitable for describing the symmetry of the distribution.

Nonparametric copula models offer greater flexibility as they do not rely on previous knowledge or assumptions about known distributions. However, there are only a few studies that have considered the nonparametric copula approach for analyzing directional wind energy. Therefore, this paper aims to explore and describe the nonlinear correlation mechanism between wind vector variables employing this model. The methodology introduced by Charpentier et al. [33] involves utilizing the transformation method to construct a nonparametric copula model, which can effectively avoid the boundary error of kernel estimation. In this paper, Gaussian CDF Φ is selected as the transformation function; given the variable (u,v), the transformed binary variable is $\left(s,t\right)=\left(\Phi^{-1}\left(u\right),\Phi^{-1}\left(v\right)\right)$. Depending on Sklar’s theorem, the joint density function of the correlated variables can be derived as follows:

(21) f(s,t)=c(Φ(s),Φ(t))ϕ(s)ϕ(t)

where Φ denotes the standard Gaussian CDF and *φ* is the first-order derivative of Φ. Hence, the above equation can be estimated using standard kernel density methods to obtain an estimate of $\hat{f}(s,t)$. In turn, the estimation of the copula density for wind speed and direction can be obtained:

(22) $$\hat{c}(u,v)=\frac{\hat{f}\left(\Phi^{-1}(u),\Phi^{-1}(v)\right)}{\phi \left(\Phi^{-1}(u)\right)\phi \left(\Phi^{-1}(v)\right)},\;(u,v)\in {\left[0,1\right]}^{2}$$

The MISE criterion and CV criterion are still employed when determining the bandwidth, with their expressions as follows:

(23) $$MISE=Ε\left\{\int {\left(\hat{c}(u,v)-c(u,v)\right)}^{2}w\left(u,v\right)dudv\right\}$$

(24) $$\mathrm{CV}(h,\theta )=\int {\left(\hat{c}(u,v)\right)}^{2}w\left(u,v\right)dudv-\frac{2}{n}\overset{n}{\underset{i=1}{\sum }}{\hat{c}}^{\left(-i\right)}\left({\hat{U}}_{i},{\hat{V}}_{i}\right)w\left({\hat{U}}_{i},{\hat{V}}_{i}\right)$$

in which $w\left(m,n\right)=\phi \left(\Phi^{-1}(m)\right)\phi \left(\Phi^{-1}(n)\right)$, ${\hat{c}}^{\left(-i\right)}\left({\hat{U}}_{i},{\hat{V}}_{i}\right)$ is the $\hat{c}$ excluding the *i*th observation at the estimation of $\left({\hat{U}}_{i},{\hat{V}}_{i}\right)$.

Two optimal smoothing parameter selection methods, i.e., the PI algorithm and the profile CV algorithm, are applied building upon the improved transform kernel estimation method [47]. The optimal bandwidth for the non-parametric copula is calculated by minimizing Equations (23) and (24). The JPDF between the two variables of wind vector can be calculated by estimating $\hat{c}(u,v)$. These two non-parametric copula-based models are referred to as KDE-COP-PI and KDE-COP-CV. Additionally, several parametric copula models are introduced in this paper for comparison.

## 3. Wind Data

**Figure 1:**
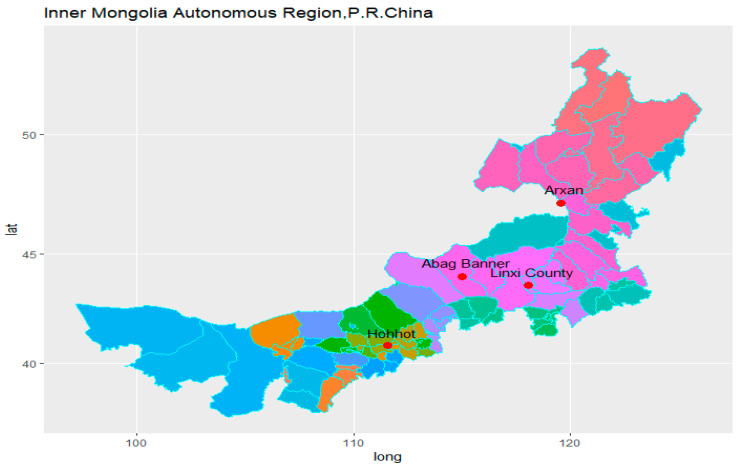
Location of four selected stations in Inner Mongolia Autonomous Region, China.

In this study, the actual observed data of daily maximum wind speed and the corresponding wind direction of four meteorological stations in the Inner Mongolia Autonomous Region of China were used as the samples. These four meteorological sites located in central and eastern Inner Mongolia play a vital role in regional climate monitoring, and their geographic information is provided in Table 2. The location information is shown in Figure 1. Subsequently, for the sake of brevity in the article, the stations Hohhot, Arxan, Abag Banner, and Linxi County will be referred to as S1, S2, S3, and S4 in that order. Wind data available taken from the China National Weather Network for the period 5 years were used in this paper. The wind direction belongs to cyclic data, which takes values from 0° to 360°, with 0° being the due north direction, clockwise as positive, and 22.5° as the interval, for a total of 16 directions, which are named due north, north-northeast, northeast, east-northeast, due east, east-southeast, southeast, south-southeast, due south, south-southwest, southwest, west-southwest, due west, west-northwest, northwest, and north-northwest in order and are noted as N, NNE, NE, ENE, E, ESE, SE, SSE, S, SSW, SW, WSW, W, WNW, NW, NNW, respectively.

## 4. Results and Discussions

### 4.1. Fitting Results for Marginal Distributions

**Figure 2:**
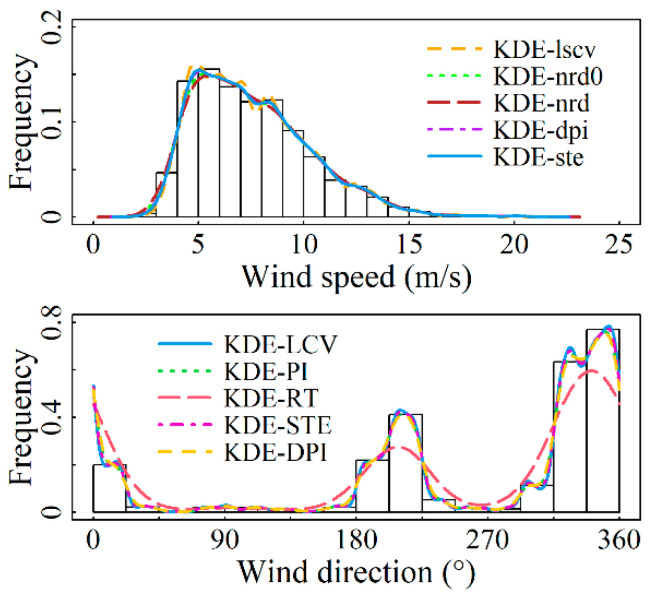
Marginal PDFs fitting results for Site S1 using various models.

**Figure 3:**
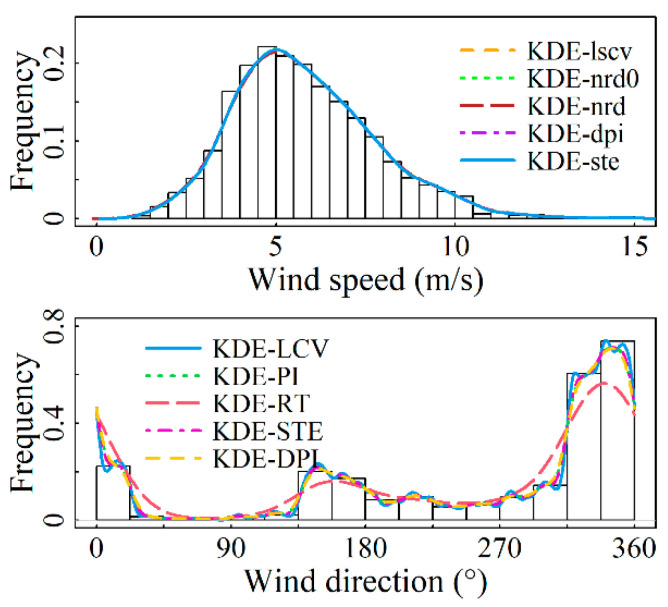
Marginal PDFs fitting results for Site S2 using various models.

**Figure 4:**
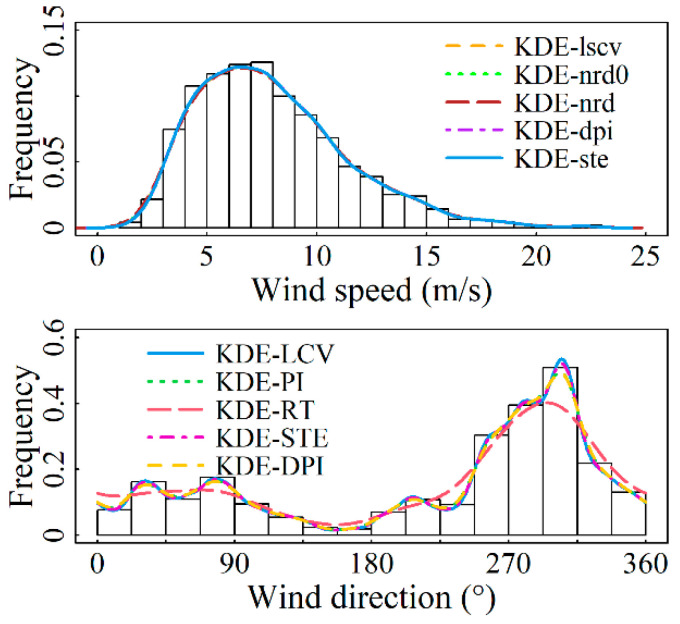
Marginal PDFs fitting results for Site S1 using various models.

**Figure 5:**
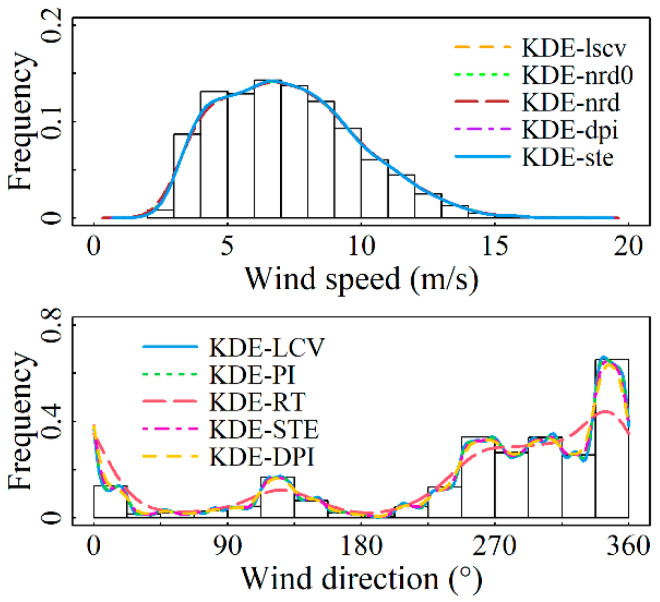
Marginal PDFs fitting results for Site S2 using various models.

For the marginal distributions of wind speed and direction, the bandwidth values and assessment metrics values calculated by using the nonparametric kernel density estimation model presented in Section 2 for each of the four sites are shown in Table 3 and Table 4. From Table 3, it is demonstrated that the solve-the-equation plug-in rule (KDE-ste) has an excellent fit at all four sites. In particular, this model outperforms other models in terms of evaluation criteria at the Abag Banner and Linxi County observation stations, ranking second in evaluation metrics at Hohhot and Arxan. Although the model’s fitting goodness for these two stations is not the best, their $R^{2}$-values reach 0.99981 and 0.99969, respectively, with both error indicators having an accuracy of 1 × 10^−3^. This level of fitting goodness is achieved, which most parameter models fail to reach. Table 4 indicates that for the modeling of wind direction distribution at the four sites, the ${\mathrm{KDE}-}_{\mathrm{LCV}}$ model demonstrates the optimal fitting accuracy, with the $R^{2}$-value reaching a maximum of 0.9999. Figure 2, Figure 3, Figure 4 and Figure 5 display the results of fitting marginal PDFs of wind vectors.

In summary, model KDE-ste and Model ${\mathrm{KDE}-}_{\mathrm{LCV}}$ are separately chosen as the wind speed and direction distribution models for the four stations under study. The corresponding *h* values are 0.42104, 0.40729, 0.65195, and 0.49787 for wind speed, while ν values of 301.5840, 352.1935, 96.7885, and 334.0411 are for wind direction, respectively.

### 4.2. Results of Fitting the Joint Probability Density Function

In the context of two non-parametric copula models, KDE-COP-PI and KDE-COP-CV, their bandwidth parameters are derived by Equations (23) and (24). Additionally, a semi-parametric estimation method is accessed to determine the parameters of the aforementioned five parameter copula models. To validate and compare the proposed models, the RMSE presented in Table 1 is utilized to assess the error metric of the JPDF, while a novel metric, namely the index of agreement (IA), is introduced to assess the degree of appropriateness of the JPDF’s fit. The IA is defined as follows:

(25) $$IA=1-\frac{\sum_{i,j}{\left(F_{ij}-{\hat{F}}_{ij}\right)}^{2}}{\sum_{i,j}{\left(\left|F_{ij}-{\bar{F}}_{ij}\right|+\left|{\hat{F}}_{ij}-{\bar{\hat{F}}}_{ij}\right|\right)}^{2}}$$

where $F_{ij}$ and ${\hat{F}}_{ij}$ denote the actual distribution value and estimated theoretical distribution value at (*i*, *j*), respectively; ${\bar{F}}_{ij}$ and ${\bar{\hat{F}}}_{ij}$ represent the average values of $F_{ij}$ and ${\hat{F}}_{ij}$, respectively.

**Figure 6:**
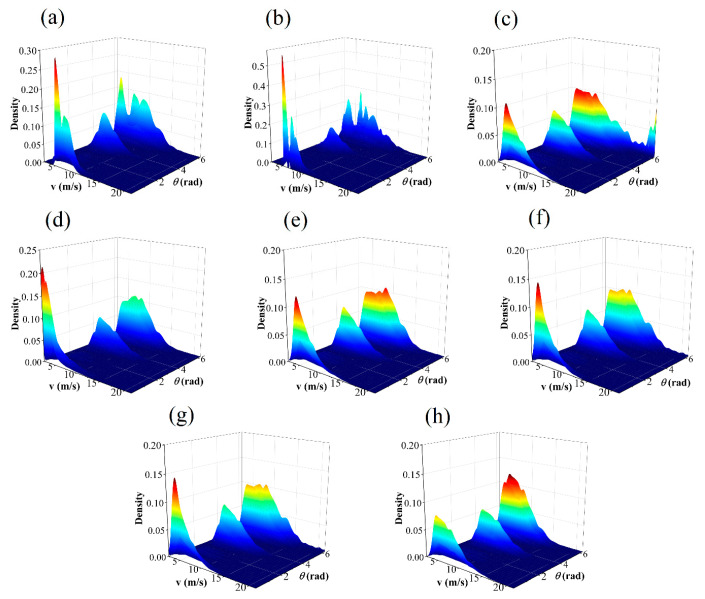
JPDF plots of node S1 using various models: (**a**) KDE–COP–PI; (**b**) KDE–COP–CV; (**c**) Gumbel; (**d**) Clayton; (**e**) Frank; (**f**) Gaussian; (**g**) Student t; (**h**) Independent of wind speed and direction.

**Figure 7:**
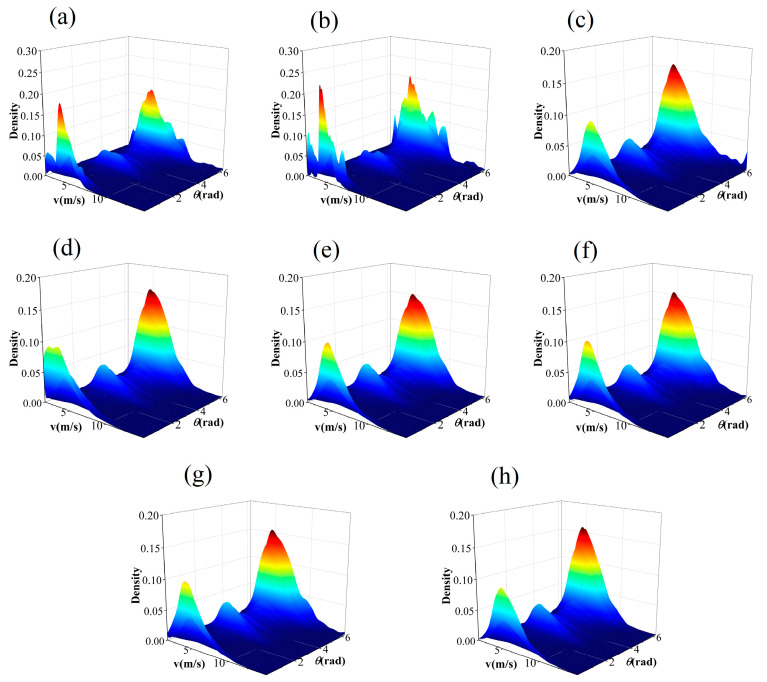
JPDF plots of node S2 using various models: (**a**) KDE–COP–PI; (**b**) KDE–COP–CV; (**c**) Gumbel; (**d**) Clayton; (**e**) Frank; (**f**) Gaussian; (**g**) Student t; (**h**) Independent of wind speed and direction.

**Figure 8:**
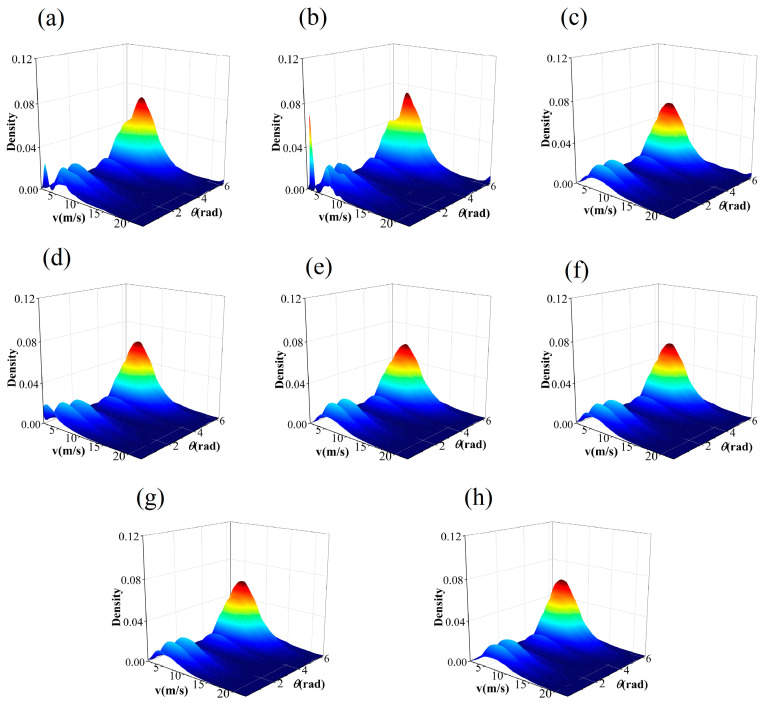
JPDF plots of node S3 using various models: (**a**) KDE–COP–PI; (**b**) KDE–COP–CV; (**c**) Gumbel; (**d**) Clayton; (**e**) Frank; (**f**) Gaussian; (**g**) Student t; (**h**) Independent of wind speed and direction.

**Figure 9:**
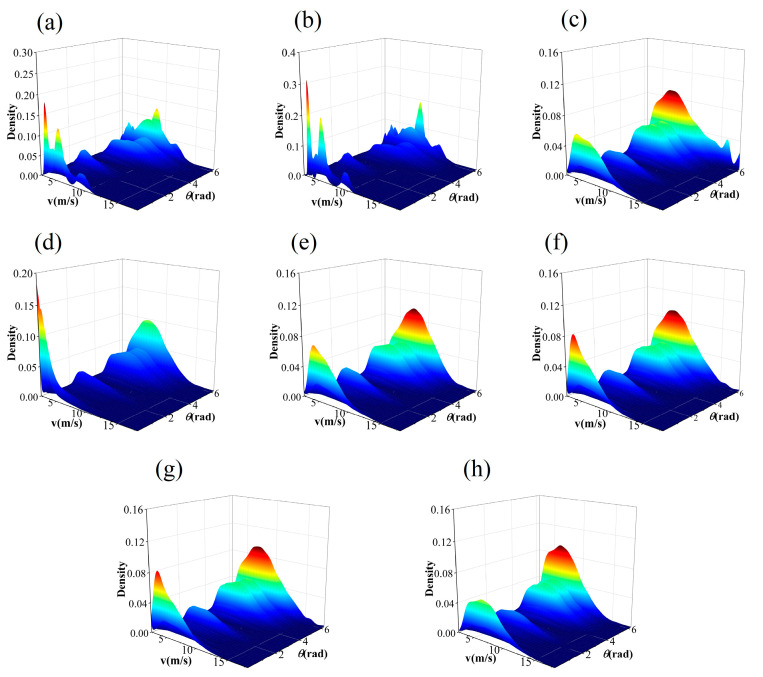
JPDF plots of node S4 using various models: (**a**) KDE–COP–PI; (**b**) KDE–COP–CV; (**c**) Gumbel; (**d**) Clayton; (**e**) Frank; (**f**) Gaussian; (**g**) Student t; (**h**) Independent of wind speed and direction.

The IA takes values between 0 and 1. The closer the value is to 1, the better the specified distribution fits the observed value data. The JPDF fitting results of seven models are illustrated in Figure 6, Figure 7, Figure 8 and Figure 9. These results indicate that the distribution curves fitted by the parametric copula models are smoother, whereas the non-parametric copula models exhibit multiple fluctuations besides the main peak. This suggests nonparametric copula models are more adept at capturing the variabilities in wind speed and direction. Furthermore, take site S1, for example, the actual observational data show that 60% of the wind comes from the northwest and north directions, with the maximum wind speed being less than 4m/s in 5% of cases. This is consistent with Figure 6a,b. However, in the north direction, cases with a maximum wind speed of less than 4m/s account for 0%, which significantly differs from other graphics in Figure 6. The Shapiro–Wilk test for significance on the maximum wind speed in the northwest direction yields a *p*-value far less than 0.05, indicating that the wind speed in this direction does not follow or approximates a unimodal normal distribution, which is highly inconsistent with Figure 6h. These results demonstrate that non-parametric copula models offer a suitable and powerful approach for analyzing dependence structures in wind vectors. Table 5 presents the model evaluation metrics for the joint distribution of wind vector. It is evident that the performance of nonparametric copula models is superior to that of parametric copula models, corroborating the findings of Han et al. [35], where the KDE-COP-CV model exhibits optimum adaptation accuracy and robustness.

## 5. Directional Wind Energy Assessment

Wind Power Density (WPD) is a crucial metric in wind energy assessment [48]. Its formula is expressed as follows:

(26) $$WPD=\frac{1}{2}\rho v^{3}$$

In this paper, based on the constructed bivariate distribution model KDE-COP-CV and combining with Formula (26), the WPD related to wind direction is rewritten as follows:

(27) $$WPD\left(\theta \right)=\frac{1}{2}\rho \int_{0}^{\infty }x^{3}\hat{f}\left(x,\theta \right)dx$$

here, *ρ* represents air density, typically assumed to be a constant $\rho =1.225\;\mathrm{kg}/m^{3}$.

The WPD for 16 wind directions is obtained using Formula (27). The overall WPD ($\bar{\mathrm{WPD}}$) for all directions is derived by substituting the wind speed marginal PDF in place of the JPDF in Equation (27), namely

(28) $$\bar{WPD}=\frac{1}{2}\rho \int_{0}^{\infty }x^{3}\hat{f}\left(x\right)dx$$

**Figure 10:**
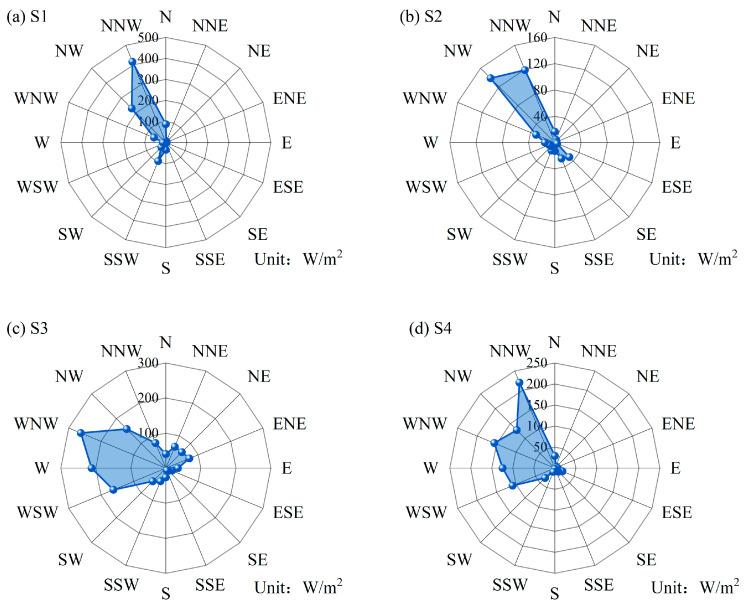
Direction-related WPD distributions using the KDE-COP-CV model at four sites.

**Figure 11:**
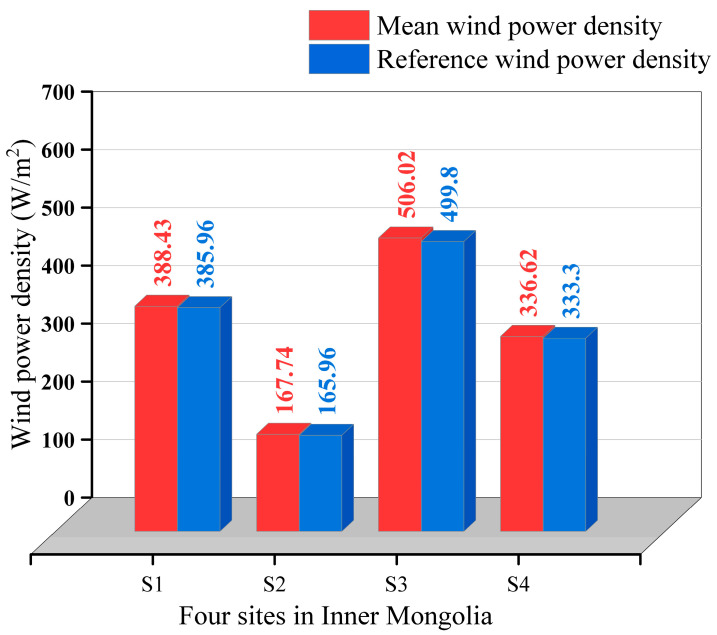
Mean $\bar{\mathrm{WPD}}$ and reference $WPD_{ref}$ at four different sites.

The reference WPD calculated using real data is denoted as $WPD_{ref}=\frac{1}{2n}\rho \sum_{i=1}^{n}x_{i}^{3}$. Table 6 compares the distribution of $\bar{\mathrm{WPD}}$ and WPD in different directions, showing significant variability in wind energy across different wind directions. Figure 10 displays the distribution of WPD across distinct wind directions. As shown in Figure 10a,b, the dominant wind directions at sites S1 and S2 are northwest and north-northwest, in which the highest WPD of S1 station reached 416.15 W/m^2^. Moreover, the WPD at site S1 in the true north, west-northwest, and south-southwest directions also exceeds 60 W/m^2^. For the S3 site, it can be seen from Figure 10c that the western, northerly, and southerly directions are more abundant, with a WPD greater than 150 W/m^2^, while the north-northeast and easterly directions have a more uniform distribution of WPD, with values ranging from 64 W/m^2^ to 73 W/m^2^. As shown in Figure 10d, the wind resources at S4 are concentrated in five directions from west-southwest to north-northwest, and the WPD values are distributed in the range of 108 W/m^2^–221 W/m^2^. To validate the fitting performance of the model employed in this study, Figure 11 illustrates the results for $\bar{\mathrm{WPD}}$ and $WPD_{ref}$ at four different sites. The WPD obtained in this paper is calculated according to the wind direction orientation of the maximum wind speed, which provides a basis for siting the turbine and determining the direction of blade rotation. If the actual turbine installation direction deviates from the direction of maximum wind speed, the obtained WPD will be overestimated.

It should be noted that this study evaluates wind energy variation based on wind speeds at 10 m above ground level. The specific wind energy density should be determined based on the hub height of different wind turbines. See https://en.wind-turbine-models.com/ (accessed on 10 December 2023) for pertinent technical parameters and power curves of the turbine model. Undoubtedly, higher hub heights correspond to greater WPD values. Differences in wind power generation potential underscore the need for directional investigations. These findings aid in optimizing the design and state monitoring of wind turbine assemblies and have significant practical implications for the design and selection of flexible structures.

## 6. Conclusions

This paper presents a nonparametric copula model on the basis of transformation methods for the analysis of wind speed and direction correlations in certain areas of Inner Mongolia. The wind speed component of the model is estimated using Gaussian kernel density, while the wind direction component is estimated using the von Mises kernel density. The five parametric copula models were introduced for model comparison, and subsequent to assessing the robustness and goodness of fit of the joint distribution models, the WPD distribution in each direction was explored based on the KDE-COP-CV model. The research conclusions are summarized as follows:

(1) For the wind speed component, the Gaussian kernel density function and five bandwidth algorithms were used to compare fitting results, with the KDE-ste model showing the best performance. For the wind direction component, the von Mises kernel density function and five circular data bandwidth algorithms were employed, with the KDE-_LCV_ model demonstrating superior fitting accuracy.
(2) The ranking results of the two evaluating metrics suggest that the performance of the two non-parametric copula models surpasses that of the parametric copulas. Among them, KDE-COP-CV outperforms KDE-COP-PI, indicating the optimum performance of the KDE-COP-CV model in modeling the JPDF of wind speed and wind direction.
(3) Based on the KDE-COP-CV model, the WPD distribution of four sites was investigated. The findings reveal that all four sites possess abundant wind resources, especially the sites located in Hohhot city (S1) and Xilin Gol League (S3). Additionally, by intuitively and accurately analyzing the change of WPD relative to the wind direction, this paper identifies that the wind energy is more substantial in the north-east and west directions. In contrast, the wind energy is considerably lower in the southeast direction. These observations highlight deviations from expected patterns when not considering dependence between wind speed and direction. The concentration range of wind energy density varies across different locations, while the directional shifts in wind energy underscore the essential need for a joint analysis involving the two variables of the bivariate wind vector.

These conclusions may have guiding significance for structural design, material selection, and wind energy assessment in some important engineering projects. Assessing dominant wind directions and directional variations in wind energy can lead to better choices in wind farm siting, as well as optimizing wind turbine capacity and spatial arrangement. The findings provide new methods for wind parameter modeling and wind energy development in other regions. Due to the power of intelligent algorithms, numerous scholars have conducted in-depth studies on wind power prediction using various artificial intelligence algorithms, making it an effective and cutting-edge alternative [49,50]. Furthermore, to more accurately represent wind energy, consideration of multi-variable joint probability density modeling, including wind speed, wind direction, wind shear, and air density, is necessary. This will be the subject of further research.

## Figures and Tables

**Table 1:** Metrics for assessing the accuracy of fit.

Metrics	Formulas
$R^{2}$	$R^{2}=1-\frac{\sum_{i=1}^{n}{\left(F_{i}-{\hat{F}}_{i}\right)}^{2}}{\sum_{i=1}^{n}{\left(F_{i}-\bar{F}\right)}^{2}}$
RMSE	$RMSE=\sqrt{\frac{1}{n}\sum_{i=1}^{n}{\left(F_{i}-{\hat{F}}_{i}\right)}^{2}}$
MAE	$MAE=\frac{1}{n}\sum_{i=1}^{n}\left|F_{i}-{\hat{F}}_{i}\right|$

**Table 2:** Geographic location for the four observatory sites.

Station	Affiliated League	Longitude (E)	Latitude (N)	Elevation (m)
Hohhot	Hohhot City	$111{}^{\circ}57^{\prime }$	40°86′	1153.5
Arxan	Hinggan League	$119{}^{\circ}93^{\prime }$	47°18′	997.0
Abag Banner	Xilin Gol League	$115{}^{\circ}00^{\prime }$	$44{}^{\circ}02^{\prime }$	1147.7
Linxi County	Chifeng City	$118{}^{\circ}03^{\prime }$	$43{}^{\circ}63^{\prime }$	825.0

**Table 3:** Estimated bandwidth and evaluation results for wind speed models at various sites.

		KDE-lscv	KDE-nrd0	KDE-nrd	KDE-dpi	KDE-ste
S1	h	0.25306	0.56485	0.66527	0.45095	0.42104
	$R^{2}$	0.99995	0.99957	0.99929	0.99978	0.99981
	RMSE	0.00206	0.00599	0.00768	0.00433	0.00393
	MAE	0.00167	0.00434	0.00554	0.00322	0.00296
S2	h	0.42174	0.39584	0.46621	0.40870	0.40729
	$R^{2}$	0.99966	0.99972	0.99952	0.99952	0.99969
	RMSE	0.00534	0.00483	0.00629	0.00508	0.00505
	MAE	0.00447	0.00398	0.00538	0.00422	0.00419
S3	h	0.70073	0.68510	0.80690	0.66153	0.65195
	$R^{2}$	0.99968	0.99970	0.99950	0.99973	0.99974
	RMSE	0.00519	0.00502	0.00647	0.00477	0.00467
	MAE	0.00431	0.00417	0.00536	0.00397	0.00388
S4	h	0.53944	0.52687	0.62053	0.51423	0.49787
	$R^{2}$	0.99979	0.99980	0.99966	0.99982	0.99983
	RMSE	0.00421	0.00406	0.00529	0.00391	0.00372
	MAE	0.00328	0.00317	0.00412	0.00306	0.00292

**Table 4:** Estimated bandwidth and evaluation results for wind direction models at various sites.

		${\mathrm{KDE}-}_{\mathrm{LCV}}$	${\mathrm{KDE}-}_{\mathrm{RT}}$	${\mathrm{KDE}-}_{\mathrm{PI}}$	${\mathrm{KDE}-}_{\mathrm{DPI}}$	${\mathrm{KDE}-}_{\mathrm{STE}}$
S1	ν	301.5840	11.6319	135.9957	119.4548	209.1291
	$R^{2}$	0.9966	0.9274	0.9931	0.9922	0.9953
	RMSE	0.01686	0.07772	0.02402	0.02549	0.01980
	MAE	0.01657	0.07169	0.02345	0.02483	0.01941
S2	ν	352.1935	10.4348	110.5933	89.7135	135.5769
	$R^{2}$	0.9981	0.9378	0.9943	0.9930	0.9953
	RMSE	0.01242	0.07198	0.02174	0.02406	0.01970
	MAE	0.01212	0.06634	0.02110	0.02331	0.01915
S3	ν	96.7885	8.8751	51.0091	49.2768	77.9118
	$R^{2}$	0.9999	0.9964	0.9997	0.9996	0.9998
	RMSE	0.00347	0.01741	0.00534	0.00546	0.00402
	MAE	0.00257	0.01464	0.00404	0.00414	0.00299
S4	ν	334.0411	12.8692	257.1817	102.3456	168.9882
	$R^{2}$	0.9987	0.9593	0.9982	0.9952	0.9972
	RMSE	0.01053	0.05817	0.01217	0.02000	0.01528
	MAE	0.01027	0.05530	0.01186	0.01939	0.01487

**Table 5:** The evaluation metric values for the joint distribution model of wind speed and wind direction at various sites.

	**S1**		**S2**		**S3**		**S4**	
**Model**	RMSE	IA	RMSE	IA	RMSE	IA	RMSE	IA
KDE–COP–PI	0.00822	0.99976	0.00587	0.99982	0.00531	0.99987	0.00594	0.99983
KDE–COP–CV	0.00787	0.99978	0.0056	0.99984	0.00513	0.99988	0.00531	0.99987
Gumbel	0.01648	0.99901	0.01351	0.99907	0.0122	0.99931	0.02222	0.99772
Clayton	0.02289	0.99802	0.01096	0.99938	0.01008	0.99952	0.01463	0.99898
Frank	0.01734	0.99888	0.01141	0.99933	0.00823	0.99968	0.0157	0.99885
Gaussian	0.0176	0.99885	0.01157	0.99932	0.00983	0.99955	0.01782	0.99852
Student t	0.01762	0.99885	0.01153	0.99932	0.00983	0.99955	0.01771	0.99854

**Table 6:** WPD (W/m^2^) in all wind directions and different wind directions.

Range	S1	S2	S3	S4
N	87.32	16.48	40.68	29.01
NNE	6.36	4.61	66.43	4.56
NE	0.84	0.85	64.68	3.89
ENE	4.18	0.60	72.74	4.81
E	3.88	2.18	34.35	7.23
ESE	2.63	3.85	19.29	19.68
SE	1.11	31.42	10.59	11.87
SSE	3.58	26.55	7.39	3.87
S	34.70	12.83	26.31	2.09
SSW	95.06	13.37	40.47	9.58
SW	30.31	8.22	52.78	32.95
WSW	7.69	9.51	161.85	108.57
W	13.83	15.28	211.99	124.44
WNW	61.24	30.70	263.00	156.13
NW	229.69	138.39	157.67	127.80
NNW	416.15	119.37	77.84	220.47
$\bar{\mathrm{WPD}}$	388.43	167.74	506.02	336.62
WPD	385.96	165.96	499.80	333.295

## Data Availability

This study utilized ground-based basic meteorological data from the China Meteorological Data Service Centre, which is available online at: https://data.cma.cn/data/detail/dataCode/A.0012.0001.html (accessed on 29 May 2024).
